# Myocardial Infarction Without Obstructive Coronary Disease in Later Life: Insights from the Malmö Diet and Cancer Study

**DOI:** 10.3390/jcm15155917

**Published:** 2026-07-29

**Authors:** Behzad Banihashemi, Fabrizio Ricci, Theodoros Intzilakis, Viktor Hamrefors, Hannes Holm, Artur Fedorowski

**Affiliations:** 1 Department of Clinical Sciences, Lund University, 205 02 Malmö, Sweden; behzadbanihashemi@hotmail.com (B.B.);; 2 Department of Cardiology, Skåne University Hospital, 205 02 Malmö, Sweden; 3 Department of Neuroscience, Imaging and Clinical Sciences, “G.d’Annunzio” University of Chieti-Pescara, Via dei Vestini 33, 66100 Chieti, Italy; fabrizio.ricci@unich.it; 4 University Cardiology Division, Heart Department, SS. Annunziata Polyclinic, Via dei Vestini, 66100 Chieti, Italy; 5 Department of Cardiology, Karolinska University Hospital, 171 76 Stockholm, Sweden; 6 Department of Medicine, Solna, Karolinska Institute, 171 76 Stockholm, Sweden

**Keywords:** myocardial infarction with non-obstructive coronary arteries, MINOCA, acute myocardial injury, acute coronary syndromes, geriatric cardiology

## Abstract

**Background**: The prognostic significance of myocardial infarction with non-obstructive coronary arteries (MINOCA) in older adults remains uncertain, particularly in comparison with revascularized acute coronary syndromes (ACS) and other acute myocardial injury phenotypes. **Methods**: In the prospective population-based cohort of the Malmö Diet and Cancer Study (n = 30,466), 925 participants who experienced an incident coronary event between 2009 and 2018 were classified as revascularized ACS, MINOCA, or other acute myocardial injury phenotypes. All-cause mortality was assessed using Kaplan–Meier estimates and Cox proportional-hazards models. **Results**: Of 925 patients (mean age, 81 ± 7 years; female sex, 57%), 136 (14.7%) had a non-revascularized event, including 49 (36%) classified as MINOCA and 87 (64%) as other acute myocardial injuries. All non-revascularized cases underwent detailed review of angiographic and clinical records. During a median follow-up of 2.5 years, mortality was substantial across all groups (81%, overall). In unadjusted analyses, survival did not differ significantly among event categories (*p* = 0.302). In multivariable analysis, MINOCA was associated with increased mortality compared with revascularized ACS (aHR 1.48; 95% CI 1.07–2.05; *p* = 0.018) and with other acute myocardial injury phenotypes (aHR 1.44; 95% CI 1.01–2.04; *p* = 0.041). Other acute myocardial injury phenotypes did not differ significantly from revascularized ACS (aHR 1.03; 95% CI 0.78–1.35). **Conclusions**: MINOCA is associated with higher mortality than both revascularized ACS and other acute myocardial injury phenotypes. Despite the absence of obstructive coronary disease, MINOCA in later life carries an adverse prognosis and commands systematic diagnostic evaluation, careful phenotypic characterization, and appropriate risk-directed management.

## 1. Introduction

Myocardial infarction with non-obstructive coronary arteries (MINOCA) represents a clinically heterogeneous entity occurring in the absence of obstructive epicardial coronary disease [1]. Although it accounts for a minority of presentations among patients with acute coronary syndromes, recognition of MINOCA has increased with routine coronary angiography and high-sensitivity cardiac troponin testing. The mechanisms underlying MINOCA are diverse and may involve coronary plaque disruption without persistent obstruction, coronary vasomotor abnormalities, microvascular dysfunction, myocardial inflammation, stress-induced cardiomyopathy, or other non-atherothrombotic processes [2,3]. This biological heterogeneity has contributed to diagnostic uncertainty and ongoing debate regarding prognosis and optimal management. Accordingly, MINOCA should be regarded as a working diagnosis that requires further etiologic clarification rather than as a final diagnosis [1], an issue that is particularly relevant in older adults in whom competing cardiac and systemic diagnoses are frequent.

Reported outcomes in MINOCA have been inconsistent. Some studies have suggested a more favorable prognosis compared with obstructive acute coronary syndromes [4,5,6,7,8,9], whereas others have demonstrated similar [5,10,11] or even higher mortality [12]. Variability in definitions, diagnostic work-up, and case ascertainment, together with differences in comparator groups, has complicated interpretation of existing data. Notably, few studies have examined whether MINOCA carries risk distinct not only from revascularized acute coronary syndromes but also from other acute myocardial injury phenotypes that do not undergo revascularization.

The uncertainty is magnified in older adults frequently presenting with multimorbidity, structural heart disease, atrial arrhythmias, and frailty. In this population, myocardial injury may represent the interplay of competing cardiac and systemic processes. Whether MINOCA in later life reflects a relatively benign manifestation of myocardial injury in the absence of obstructive disease, or instead identifies a subgroup at heightened risk, remains unclear. Older individuals are underrepresented in many contemporary MINOCA cohorts despite accounting for a substantial proportion of hospital admissions for myocardial injury. The Malmö Diet and Cancer (MDC) Study provides a well-characterized, population-based framework [13] in which to examine the prognostic relevance of MINOCA in older adults. In this study, we aimed to characterize older patients with MINOCA and to assess their all-cause mortality compared with patients with revascularized ACS and other non-revascularized acute myocardial injury phenotypes. This comparison was intended to address whether MINOCA in later life represents a comparatively low-risk presentation in the absence of obstructive epicardial coronary disease or instead identifies a clinically meaningful high-risk condition.

## 2. Methods

### 2.1. Study Population

The study population was derived from the MDC Study, a prospective, population-based cohort conducted in Malmö, Sweden. Between 1991 and 1996, adults born between 1923 and 1950 were invited to participate [13]. A total of 30,466 individuals underwent baseline examination, of whom 28,098 provided complete dietary, questionnaire, and anthropometric data. All participants provided written informed consent. The study was approved by the Regional Ethical Review Board at Lund University (approval number LU 51/90). All study participants were followed up through 31 December 2018 by linking their unique 10-digit personal identification number with the Swedish National Hospital Discharge Register (SNHDR), Swedish National Cause of Death Register (SNCDR), and Stroke Register of Malmö (STROMA).

Because there is no specific ICD-10 code for MINOCA, we established a pragmatic case-identification strategy based on diagnostic and procedural coding, followed by clinical adjudication. The MDC cohort was linked to national and regional hospital registers to identify all ischemic heart disease events (ICD-10 codes I20–I24). We identified 4033 incident coronary events during follow-up. The register-based diagnosis of coronary event in SNHDR has been found to be highly valid [14]. To ensure homogeneity of biomarker data, we restricted analyses to events occurring after the implementation of high-sensitivity cardiac troponin testing in Sweden from 2009 onward. This yielded 925 participants with incident acute myocardial injury between 2009 and 2018. Of these, 136 patients (14.7%) had a non-revascularized event. All these cases underwent detailed review of angiographic and clinical records by a cardiologist (B.B.). Events were classified using procedural codes, coronary angiography data (AF037), discharge diagnoses, biomarker information, and clinical records. Individuals with evidence of revascularization, either percutaneous coronary intervention (Z95.5) or coronary artery bypass grafting (Z95.1), were classified as revascularized acute coronary syndrome (ACS). Non-revascularized cases were categorized into mutually exclusive groups: MINOCA or other non-revascularized acute myocardial injury phenotypes.

MINOCA was defined as acute myocardial infarction fulfilling the 4th Universal Definition of Myocardial Infarction, occurring in patients who underwent coronary angiography showing non-obstructive epicardial coronary artery disease, defined as no stenosis ≥ 50% in any major epicardial coronary artery, and without a clinically overt alternative diagnosis at presentation [1]. The group of other non-revascularized myocardial injury phenotypes included obstructive coronary disease not suitable for intervention, type 2 myocardial infarction or supply–demand mismatch, myocarditis, takotsubo syndrome, acute heart failure, primary arrhythmic events, hypertensive crisis or hypertension-related myocardial injury, COPD-related myocardial injury, and other non-coronary or non-ischemic acute injury mechanisms. Age was not an eligibility criterion for the present analysis. The term “older adults” is used descriptively to reflect the age distribution of the cohort at the index event.

### 2.2. Statistical Analysis

Baseline characteristics were summarized according to coronary event phenotype: revascularized ACS, MINOCA, and other acute myocardial injury phenotypes. Continuous variables are presented as mean ± standard deviation or median (interquartile range), as appropriate, and categorical variables as counts and percentages. Between-group differences were assessed using ANOVA or Kruskal–Wallis tests for continuous variables and χ^2^ tests for categorical variables. The primary endpoint was all-cause mortality after the index coronary event. Participants entered the analysis on the date of that event, which served as time zero. Follow-up continued until death or administrative censoring at the end of the study period. Unadjusted survival was estimated using the Kaplan–Meier method and compared across groups using the log-rank test. The association between event phenotype and mortality was examined using Cox proportional-hazards regression. Revascularized ACS served as the reference category. Hazard ratios (HRs) are reported with 95% confidence intervals (CIs). Covariate selection was prespecified and guided by clinical relevance and potential confounding structure rather than automated variable selection procedures. Because the MINOCA subgroup was modest in size and several index-event variables were not systematically available or harmonized across the full cohort, the primary model was intentionally parsimonious and focused on demographic characteristics and major pre-existing cardiovascular comorbidities. Given the advanced age of the cohort and the limited size of the MINOCA subgroup, a hierarchical modeling strategy was adopted to preserve parsimony and avoid overfitting. Model 1 adjusted for age and sex. Model 2 (primary model) further adjusted for major cardiovascular comorbidities, including prior heart failure and atrial fibrillation. Extended models incorporating additional comorbidities were explored in sensitivity analyses where model stability permitted. Care was taken to avoid collinearity between event phenotype and pre-existing coronary disease. Event phenotype was modeled simultaneously as a multinomial exposure, allowing estimation of risk relative to revascularized ACS. Direct contrasts between MINOCA and other myocardial injury phenotypes were derived within the same regression framework. The proportional hazards assumption was evaluated using Schoenfeld residuals and inspection of log-minus-log survival plots; no major violations were observed in the primary model. Missing covariate data were minimal (<10% for all variables included in the primary model), and complete-case analysis was performed. All tests were two-sided, with *p* values < 0.05 considered statistically significant. Statistical analyses were performed using R software (version 4.3 or later; R Foundation for Statistical Computing, Vienna, Austria).

## 3. Results

### 3.1. Characteristics of Study Population

**Figure 1:**
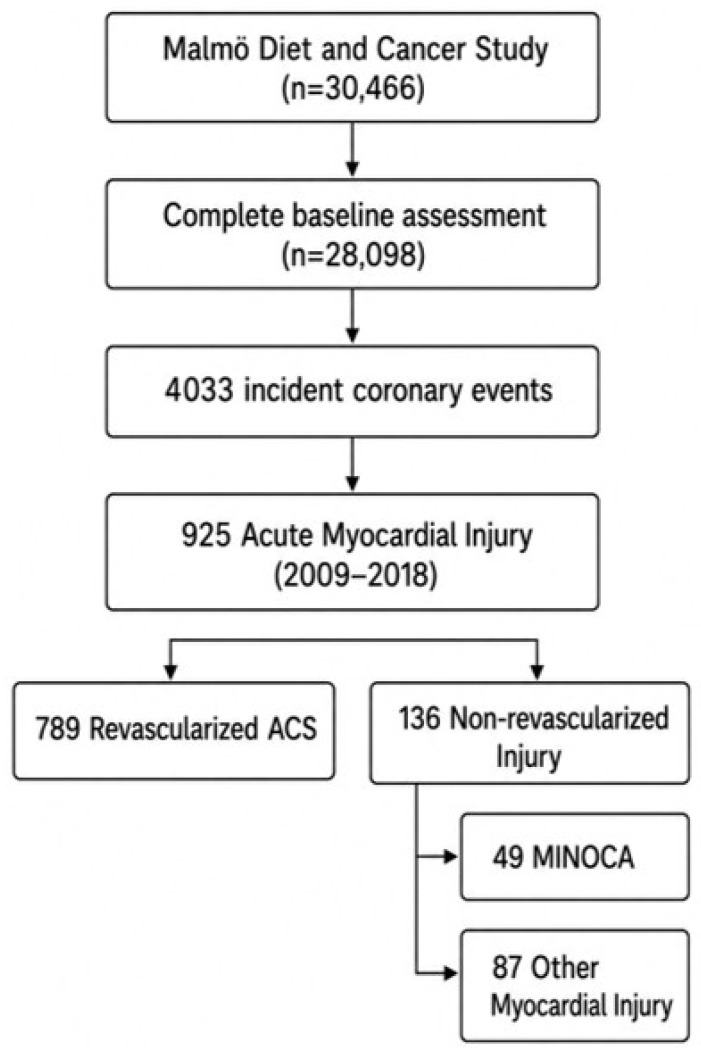
Study flow chart. Flow diagram showing the selection of participants from the Malmö Diet and Cancer Study cohort, identification of incident coronary events occurring after the implementation of high-sensitivity cardiac troponin testing, and final classification into revascularized ACS (acute coronary syndrome), MINOCA (myocardial infarction with non-obstructive coronary arteries), and other acute myocardial injury phenotypes.

A total of 925 participants experienced an incident coronary event and were included in the analysis (Figure 1). Of these, 789 (85.3%) were classified as having revascularized ACS, 49 (5.3%) as having MINOCA, and 87 (9.4%) as having other acute myocardial injury. Within the latter subgroup, obstructive ACS not amenable to revascularization accounted for 19 cases (21.8%), acute heart failure for 19 (21.8%), primary arrhythmic events for 22 (25.3%), type 2 myocardial infarction for 5 (5.7%), and other non-ischemic mechanisms for 22 (25.3%). Because of the small number of patients within each component of the other acute myocardial injury group, subgroup-specific survival analyses were not performed.

**Figure 2:**
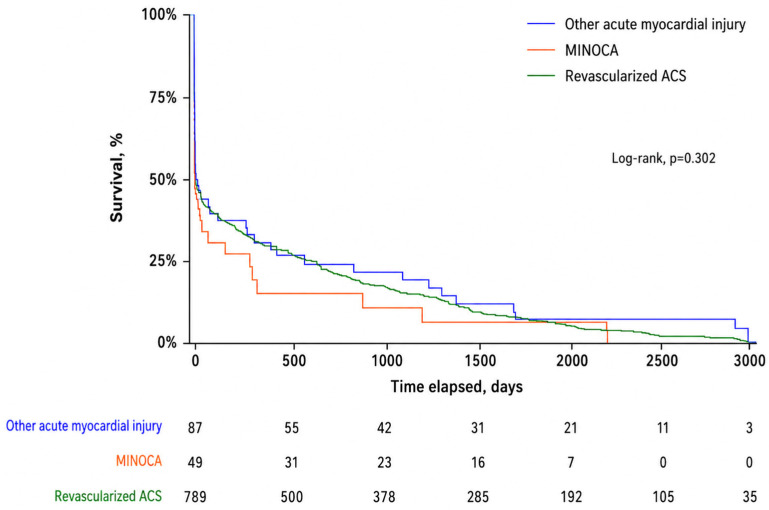
Survival analysis. Kaplan–Meier curves showing survival probability after the index coronary event among patients with revascularized acute coronary syndrome, myocardial infarction with non-obstructive coronary arteries, and other acute myocardial injury phenotypes. Survival did not differ significantly among groups in unadjusted analysis by the log-rank test. ACS, acute coronary syndrome; MINOCA, myocardial infarction with non-obstructive coronary arteries.

The mean age at baseline screening and inclusion in the MDC study was 62 ± 7 years. The mean age at the time of the index event was 81.4 ± 6.6 years in the revascularized ACS group, 80.8 ± 7.8 years in the MINOCA group, and 80.6 ± 8.7 years in the group with other causes of myocardial injury (*p* = 0.48). Hypertension and diabetes were evenly distributed across categories. The prevalence of prior coronary artery disease, stroke, heart failure, and atrial fibrillation was consistently low and showed no significant between-group differences (Table 1). Over a median post-event follow-up of 2.5 years (interquartile range, 0.4 to 5.4), calculated from the date of the index coronary event to death or administrative censoring on 31st December 2018, 750 deaths occurred (81.1% of the cohort). This proportion represents cumulative all-cause mortality during the available post-event follow-up period. Mortality was observed in 81.5% of patients with revascularized ACS, 85.7% of those with MINOCA, and 74.7% of those with other myocardial injury. Kaplan–Meier survival estimates did not differ significantly among the three phenotypes (log-rank *p* = 0.302) (Figure 2).

### 3.2. Survival Analyses

In unadjusted Cox analysis, MINOCA was not significantly associated with mortality relative to revascularized ACS (HR, 1.27; 95% confidence interval [CI], 0.93 to 1.73), nor was other myocardial injury (HR, 0.94; 95% CI, 0.72 to 1.23). After adjustment for age and sex, MINOCA was associated with a higher hazard of death (adjusted hazard ratio [aHR], 1.62; 95% CI, 1.18 to 2.23; *p* = 0.003), whereas other myocardial injury was not (aHR, 1.09; 95% CI, 0.84 to 1.41; *p* = 0.53). In the prespecified primary multivariable model, which incorporated demographic characteristics and baseline cardiovascular comorbidity, MINOCA remained independently associated with mortality (aHR, 1.48; 95% CI, 1.07 to 2.05; *p* = 0.018). Other myocardial injury was not associated with an increased hazard of death (aHR, 1.03; 95% CI, 0.78 to 1.35; *p* = 0.83). Direct comparison within the same model indicated a higher hazard of death for MINOCA than for other myocardial injury (aHR, 1.44; 95% CI, 1.01 to 2.04; *p* = 0.041). HRs and corresponding confidence intervals for all models are presented in Table 2. No substantive violations of the proportional-hazards assumption were detected on inspection of Schoenfeld residuals and log-minus-log survival curves.

## 4. Discussion

In this prospective, community-based cohort of older adults with adjudicated acute myocardial injury, MINOCA was independently associated with increased long-term all-cause mortality compared with both revascularized ACS and other non-revascularized myocardial injury phenotypes. This association persisted after adjustment for age, sex, and major cardiovascular comorbidities, whereas other non-revascularized myocardial injury phenotypes did not differ significantly from revascularized ACS. These findings suggest that, in later life, MINOCA should not be regarded as a benign or merely residual diagnostic category, but rather as a clinically meaningful high-risk phenotype despite the absence of obstructive coronary artery disease on angiography.

These findings address a persistent inconsistency in the MINOCA prognosis where previous studies have variably reported lower, similar, or even higher mortality in MINOCA compared with obstructive ACS, likely reflecting substantial heterogeneity in definitions, diagnostic work-up, comparator groups, and study populations [4,6,8,9]. Prior studies have largely contrasted MINOCA with obstructive myocardial infarction, without accounting for the heterogeneous spectrum of non-revascularized myocardial injury. This distinction is particularly important in older patients, in whom troponin elevation frequently occurs in the context of arrhythmia, heart failure, frailty, or systemic illness [15]. By directly contrasting MINOCA not only with revascularized ACS but also with other adjudicated non-revascularized myocardial injury phenotypes, our study provides a more granular estimate of risk in a clinically relevant older population. The observed pattern, in which MINOCA, but not the heterogeneous group of other non-revascularized myocardial injury phenotypes, remained independently associated with mortality, supports the clinical relevance of MINOCA in older adults. However, this comparison should be interpreted cautiously given the biological diversity and limited subgroup size of the comparator group.

Several pathophysiological mechanisms may explain this adverse prognosis. First, the absence of obstructive epicardial stenosis on angiography does not exclude clinically important coronary pathology, including plaque disruption without persistent occlusion, distal embolization, transient thrombosis, coronary spasm, spontaneous reperfusion, or diffuse atherosclerosis that remains angiographically underestimated. Second, coronary microvascular dysfunction may play a central role, particularly in women, and may contribute to myocardial infarction in the absence of flow-limiting epicardial disease [16]. Third, in older patients, myocardial ischemia may arise through a complex interaction between limited cardiovascular reserve, vascular stiffening, atrial arrhythmias, structural heart disease, and oxygen supply–demand mismatch. In such a setting, MINOCA may identify not a milder form of infarction, but rather a more heterogeneous and biologically vulnerable state in which ischemic injury occurs in the context of reduced resilience and multimorbidity.

The age profile of our cohort is likely crucial for interpreting the results. Most contemporary MINOCA cohorts are younger and include a greater proportion of patients with fewer competing risks and fewer noncoronary comorbidities [17]. In contrast, our study population had a mean age above 80 years at the index event, a setting in which frailty and the presence of comorbidities are common and may amplify the clinical consequences of MINOCA. Thus, while MINOCA may sometimes appear to carry a more favorable prognosis in younger or more selected populations, our data indicate that this assumption should not be extrapolated to older adults [17]. In later life, MINOCA may instead function as a marker of advanced cardiovascular vulnerability, helping explain why the prognostic signal became more apparent after multivariable adjustment rather than disappearing. Accordingly, the high cumulative mortality observed in the present study should be interpreted in light of this advanced age profile, the population-based design, and the use of all-cause mortality as the endpoint. In this setting, death may reflect not only the index myocardial injury phenotype but also competing risks, frailty, comorbidity burden, functional status, acute illness severity, left ventricular function, renal function, and subsequent non-cardiovascular events, which were not systematically available and may have influenced prognosis.

Another plausible explanation for the worse long-term outcome is variation in downstream management. In routine practice, patients with MINOCA may be less likely than patients with obstructive ACS to receive intensive secondary preventive therapy, partly because the absence of obstructive coronary disease may create uncertainty regarding mechanism and treatment strategy [18]. This therapeutic uncertainty is well recognized, where previous reports emphasize that MINOCA should be understood as a working diagnosis requiring further etiologic clarification rather than a final diagnosis in itself [19]. Observational data from SWEDEHEART have suggested that statins and renin–angiotensin system inhibitors are associated with improved outcomes in MINOCA, with less consistent evidence for dual antiplatelet therapy and beta-blockers [20]. It is therefore plausible that under-recognition, undertreatment, or lower treatment persistence may contribute to the adverse prognosis observed in real-world older populations.

The predominance of women in the MINOCA subgroup is consistent with previous epidemiological observations [7,21]. Sex-related differences in endothelial biology, coronary vasomotor function, plaque characteristics, and microvascular dysfunction may all contribute to the overrepresentation of women among patients with MINOCA [22,23,24]. However, adjustment for sex did not materially attenuate the association between MINOCA and mortality in our cohort, indicating that female predominance alone does not explain the adverse prognosis. Rather, sex appears to be an important feature of the phenotype, but not its sole determinant.

## 5. Strengths and Limitations

This study has several important strengths. It was conducted within a large, prospective, population-based cohort with systematic event ascertainment and near-complete follow-up through national registers. Restriction to events occurring after the implementation of high-sensitivity cardiac troponin improved diagnostic consistency, and all non-revascularized cases underwent detailed medical record review supported by procedural codes, angiography data, discharge diagnoses, biomarker information, and clinical records. This strengthens phenotypic separation and is a major advantage when studying myocardial injury syndromes in older adults, where diagnostic overlap is common.

The study also has limitations. The number of patients with MINOCA was modest, which limited precision and precluded reliable analyses according to presumed mechanism, sex, treatment exposure, or cause-specific mortality. Although the Cox models were prespecified and adjusted for clinically relevant covariates, the confidence intervals should be interpreted cautiously. In addition, cardiovascular magnetic resonance imaging, intracoronary imaging with optical coherence tomography or intravascular ultrasound, and coronary functional testing were not performed systematically. Residual misclassification between MINOCA, type 2 myocardial infarction, myocarditis, takotsubo syndrome, microvascular dysfunction, vasospasm, and plaque disruption without persistent obstruction therefore cannot be excluded. Accordingly, our findings should be interpreted as reflecting the prognosis of clinically adjudicated MINOCA in routine practice, rather than that of imaging-confirmed mechanistic MINOCA subtypes. Further, the comparator group of other non-revascularized acute myocardial injury phenotypes was heterogeneous and included obstructive ACS not amenable to revascularization, type 2 myocardial infarction or supply–demand mismatch, myocarditis, takotsubo syndrome, acute heart failure, primary arrhythmic events, hypertensive crisis or hypertension-related myocardial injury, COPD-related myocardial injury, and other non-coronary or non-ischemic mechanisms. Although this reflects real-world diagnostic complexity in older adults, the small number of patients within each subtype precluded reliable subgroup-specific survival analyses. Therefore, the comparison between MINOCA and other acute myocardial injury phenotypes should be interpreted as an exploratory comparison with a heterogeneous non-revascularized comparator group, rather than as a mechanistic comparison with individual myocardial injury subtypes. Additionally, the use of revascularized ACS as the reference group may also have introduced selection and treatment bias. Patients undergoing revascularization may differ from non-revascularized patients in frailty, coronary anatomy, comorbidity burden, procedural suitability, and intensity of secondary prevention. Moreover, successful revascularization and the standardized evidence-based treatment pathway accompanying invasive ACS management may have improved outcomes in this comparator group, potentially accentuating the relative mortality difference observed for MINOCA. Because treatment exposure, discharge therapy, medication persistence, and outcomes among comparable patients with obstructive ACS who were not revascularized were not systematically available, the contribution of revascularization itself cannot be separated from patient selection and other treatment-related differences. Accordingly, comparisons with revascularized ACS should be interpreted as prognostic comparisons with a clinically selected, invasively managed ACS phenotype rather than as evidence of a causal effect of revascularization.

These limitations should be viewed in the context of the population studied. In older adults, extensive diagnostic testing after myocardial injury is often not pursued or is considered to have limited incremental clinical value. The adverse prognostic signal observed in this cohort is therefore important because it arises precisely in a setting in which MINOCA may be under-investigated, incompletely characterized, or regarded as less actionable. Residual confounding related to frailty, comorbidity burden and functional status remains possible. Finally, several clinically relevant index-event variables, including renal function, anemia, malignancy, infection, left ventricular function, troponin magnitude, inflammatory status, and discharge therapy, were not systematically available or harmonized across the full cohort and therefore could not be incorporated into the multivariable models. In addition, cardiovascular risk factors were assessed at cohort entry rather than at the time of the index event, which may have reduced their ability to capture clinical status at presentation. Accordingly, the adjusted hazard ratios should be interpreted as adjusted prognostic associations rather than causal estimates. Further, cause-specific mortality and detailed markers of acute illness severity were not systematically available or harmonized across the full cohort. Therefore, we could not determine the relative contribution of cardiovascular and non-cardiovascular death, nor could we assess whether cardiogenic shock, inotropic or vasopressor support, or mechanical circulatory support contributed to the high observed all-cause mortality. These limitations are particularly relevant in later life with substantial competing risks. Further studies incorporating contemporary multimodality diagnostic protocols and treatment data are needed to confirm these findings and clarify the mechanisms underlying excess mortality in older patients with MINOCA.

## 6. Conclusions

Among older individuals experiencing acute myocardial injury, MINOCA was associated with increased long-term mortality compared with both revascularized ACS and other myocardial injury phenotypes. These findings indicate that MINOCA in later life is not a benign clinical entity, but rather a high-risk condition that warrants systematic diagnostic evaluation and comprehensive, etiology-informed risk management. Given the observational design, modest subgroup size, potential residual confounding, and incomplete mechanistic characterization, these findings should be considered hypothesis-generating. Larger contemporary studies incorporating frailty assessment, multimodality imaging, treatment exposure, and cause-specific outcomes are needed to confirm these observations, clarify mechanisms and improve outcomes in this growing patient population.

## Figures and Tables

**Table 1:** Baseline characteristics.

Baseline Characteristics	Overall (n = 925)	Revascularized ACS (n = 789)	MINOCA (n = 49)	Other Myocardial Injury (n = 87)	*p* Value
Age at index coronary event, years—mean ± SD	81.3 ± 6.9	81.4 ± 6.6	80.8 ± 7.8	80.6 ± 8.7	0.484
BMI, kg/m^2^—mean ± SD	26.5 ± 4.4	26.6 ± 4.4	26.3 ± 4.4	26.5 ± 4.3	0.916
Female sex—no. (%)	528 (57.1)	444 (56.3)	30 (61)	33 (37.9)	0.488
Hypertension—no. (%)	264 (28)	229 (29.0)	13 (26.5)	22 (25.3)	0.727
Diabetes mellitus—no. (%)	44 (5.7)	38 (5.8)	0 (0.0)	6 (7.9)	0.199
Current smoker—no. (%)	209 (22.5)	173 (21.9)	16 (32.6)	20 (22.9)	0.233
Dyslipidemia—no. (%)	43 (4.6)	36 (4.6)	2 (4)	5 (5.7)	0.899
Prior coronary artery disease—no. (%)	44 (4.8)	39 (4.9)	2 (4.1)	3 (3.4)	0.803
Prior stroke—no. (%)	14 (1.5)	10 (1.3)	0 (0.0)	4 (4.6)	0.036
Prior heart failure—no. (%)	3 (0.3)	3 (0.4)	0 (0.0)	0 (0.0)	0.072
Prior atrial fibrillation—no. (%)	18 (1.9)	15 (1.9)	0 (0.0)	3 (3.4)	0.366

**Table 2:** Association Between Type of Myocardial Injury Event and All-Cause Mortality in Multivariable Cox Regression.

Event Phenotype	Deaths/Total	Unadjusted HR (95% CI)	Model 1 HR (95% CI)	*p* Value	Model 2 aHR (95% CI)	*p* Value
Revascularized ACS	643/789	Ref.	Ref.	—	Ref.	—
MINOCA	42/49	1.27 (0.93–1.73)	1.62 (1.18–2.23)	0.003	1.48 (1.07–2.05)	0.018
Other myocardial injury	65/87	0.94 (0.72–1.23)	1.09 (0.84–1.41)	0.53	1.03 (0.78–1.35)	0.83

Model 1 included age at the index event and sex. Model 2 additionally included prior coronary artery disease, prior stroke, prior heart failure, and prior atrial fibrillation. aHR, adjusted hazard ratio.

## Data Availability

Data supporting the findings of this study are available from the corresponding author upon reasonable request.
